# Molecular Point-of-Care Testing for Hepatitis C: Available Technologies, Pipeline, and Promising Future Directions

**DOI:** 10.1093/infdis/jiad463

**Published:** 2023-10-28

**Authors:** Elena Ivanova Reipold, Sonjelle Shilton, Marco Donolato, Marta Fernandez Suarez

**Affiliations:** FIND, The Global Alliance for Diagnostics, Geneva, Switzerland; FIND, The Global Alliance for Diagnostics, Geneva, Switzerland; FIND, The Global Alliance for Diagnostics, Geneva, Switzerland; FIND, The Global Alliance for Diagnostics, Geneva, Switzerland

**Keywords:** hepatitis C, diagnostics, point-of-care, molecular assays

## Abstract

Hepatitis C virus (HCV) remains a major public health problem, despite the availability of effective treatments. In many areas, the ability to diagnose HCV infection at the point of care is key to scaling up access to care and treatment. To achieve this, an accurate, easy-to-use, and affordable diagnostic tool is required—this would enable decentralized testing and the creation of one-stop centers to eliminate gaps in the care cascade, which would help reach the millions of people with undiagnosed HCV infection in low- and middle-income countries and high-risk populations in high-income countries. In this review, we examine the current state of point-of-care molecular technologies, the advantages and limitations of currently available devices (both near- and true-point-of-care), the potential of molecular testing to transform diagnostic medicine in the future, and the challenges that need to be addressed for broader adoption of this technology in routine clinical practice.

Hepatitis C virus (HCV) is a blood-borne virus transmitted via direct contact with infectious blood (eg, by infusions) or indirectly via contaminated materials (eg, syringe needles or medical equipment) [1, 2]. It is recognized by the World Health Organization (WHO) as a major public health problem, with an estimated 58 million people living with chronic HCV infection worldwide and 290 000 people dying from HCV-related causes every year [3–5]. High-risk populations for HCV infection include low- and middle-income countries (LMICs), where HCV exerts a disproportionately high burden [2, 4], and groups who are regularly exposed to routes of transmission, such as people who inject drugs (PWID). Of the 15.6 million PWID between 15 and 64 years of age worldwide, it is estimated that 52%–60% are seropositive for hepatitis C [6, 7]. In addition, it is estimated that 23% of all new HCV cases and 33% of annual HCV-related deaths are among PWID [6].

With the advent of safe and potent direct-acting antiviral regimens, HCV treatment has become easier and more effective with a high cure rate (over 90% irrespective of HCV genotype and disease severity) after as little as 8 to 12 weeks of treatment [5, 8]. Despite this, and despite a 2016 WHO Global Health Sector Strategy aimed at eliminating viral hepatitis as a public health threat by 2030 [2], low treatment rates and low diagnosis rates persist—of the estimated 58 million people living with chronic HCV infection in 2019, only 21% had a confirmed diagnosis and only 13% were receiving treatment [4].

Reasons for this low uptake include (1) a lack of awareness about HCV in the population; (2) the complexity of existing diagnostic algorithms, which involve a 2-step process of screening followed by separate confirmatory testing in a centralized laboratory; (3) limited laboratory capacity in LMICs; and (4) the prohibitive costs of testing [5, 9]. As such, it is clear that the ability to diagnose HCV infection at the point of care is an important aspect of scaling up access to HCV care and treatment [5, 9]. Unfortunately, an accurate, easy-to-use, and affordable diagnostic tool to confirm an HCV diagnosis in decentralized settings is still lacking and urgently needed in LMICs to reach the large number of people with undiagnosed HCV infection [4, 5]. The availability of these tests would also help reach high-risk populations in high-income countries, and potentially enable the creation of one-stop centers to eliminate gaps in the care cascade [9, 10]. To achieve this, the ideal HCV point-of-care (POC) test would be one that (1) can be performed on capillary blood without any additional requirement for laboratory equipment; (2) is accurate (limit of detection < 3000 IU/mL and clinical sensitivity >95%); (3) integrates specimen preparation; (4) has a short turnaround time (<30 minutes); and (5) is inexpensive ($1–10 per test) [5, 11].

In this review, we will examine the current state of POC molecular testing, its advantages and limitations, and its potential to transform diagnostics and patient care in the future. We will also discuss some of the challenges that need to be addressed for broader adoption of this technology in routine clinical practice.

## TYPES OF POINT-OF-CARE DEVICES FOR MOLECULAR NUCLEIC ACID TESTING

**Figure 1.**
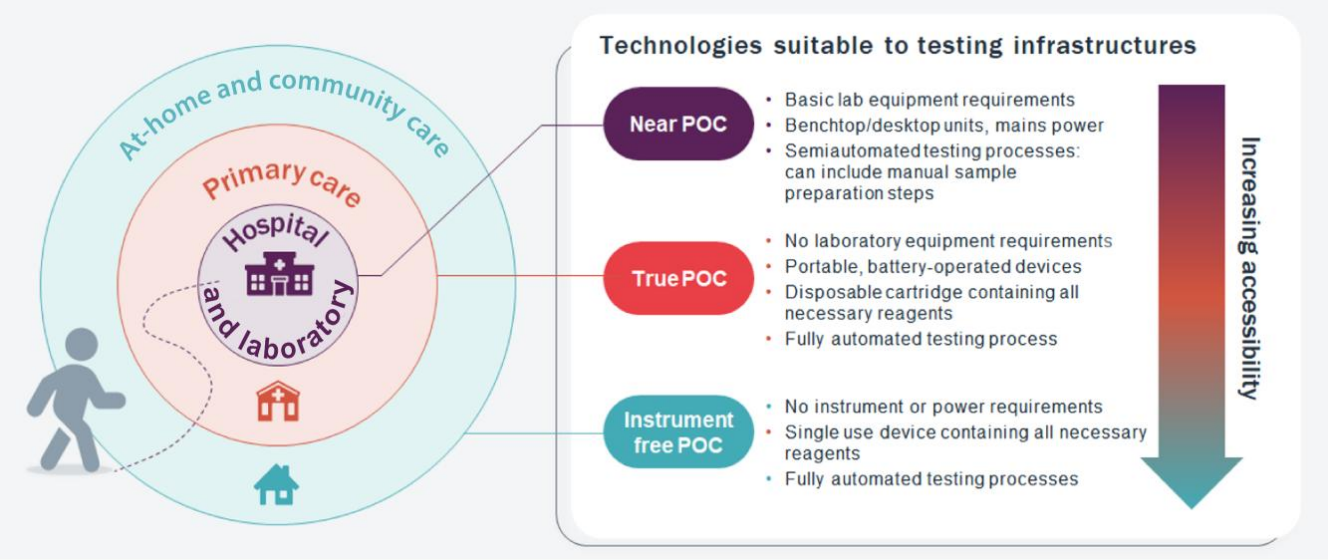
Types of point-of-care (POC) technologies.

Depending on the complexity of the design, infrastructure requirements, and ease of use, POC molecular testing platforms can be divided into 2 subcategories: near point-of-care (near-POC) platforms and true point-of-care (true-POC) platforms (Figure 1). Near-POC platforms are usually benchtop instruments that require electrical power and at least minimal laboratory infrastructure. These platforms are typically operated by experienced laboratory technicians, the testing procedures may include one or several manual steps, and operation may require additional equipment such as a centrifuge or calibrated pipettes. True-POC molecular tests are typically portable, battery-operated, integrated sample-to-answer testing solutions that require minimal training and maintenance, making them well suited for use at primary healthcare facilities. Most of the near-POC platforms utilize thermal cycling to amplify target DNA or RNA sequences (polymerase chain reaction [PCR] amplification), which requires rapid heating and cooling of the reaction mixture and hence a stable power supply to operate. PCR amplification technology provides highly accurate results; however, the complex design of the platform (which includes an instrument and a disposable assay cartridge) generally results in higher costs and infrastructure and maintenance needs. True-POC platforms are often based on isothermal amplification approaches that do not require thermal cycling, such as loop-mediated amplification (LAMP), recombinase polymerase amplification (RPA), strand displacement amplification (SDA), and other techniques [12]. Although these amplification methods do not provide the same level of accuracy as PCR, the reactions can be run at a constant temperature and testing can be performed on a simple and portable low-cost device that does not require repeated calibration and maintenance.

## NEAR POINT-OF-CARE ASSAYS FOR HCV DETECTION

The only POC tests for HCV detection currently available are on near-POC platforms (Table 1). The Cepheid GeneXpert system with *Mycobacterium tuberculosis* detection assay was the first POC molecular test endorsed by WHO in 2010. Today, the Cepheid system incorporates a wide variety of assays, including an Xpert HCV Viral Load (VL) assay and an Xpert HCV VL Fingerstick assay [13–15]. Both are sample-to-answer disposable microfluidic cartridges that contain all the necessary reagents, and the testing is run on the GeneXpert platform, a fully integrated and automated PCR-based molecular diagnostic system. The Xpert HCV VL assay is intended for use in human plasma and serum specimens with results available in 90 minutes, and the Xpert HCV VL Fingerstick assay is performed using 100 μL of fingerstick capillary blood (approximately 3 drops) and has a turnaround time of 60 minutes. The Xpert HCV VL and Xpert HCV VL Fingerstick assays have been evaluated in a number of studies, which have demonstrated the high diagnostic accuracy, usability, and acceptability of the testing platform [16–19]. A recent systematic review reported a sensitivity of 100% [95% confidence interval [CI], 98%–100%) and a specificity of 97% (95% CI, 94%–98%) for the plasma-based assay and a sensitivity of 98% (95% CI, 95%–99%) and a specificity of 99% (95% CI, 97%–99%) for the fingerstick blood-based assay [20]. Both assays have received CE-marked *in vitro* diagnostics (CE-IVD) clearance and WHO prequalification [21, 22].

**Table 1.** Currently Available Near-POC HCV Assays

	Cepheid Xpert HCV VL and Xpert HCV VL Fingerstick^a^		GeneDrive HCV ID^b^	Molbio Truenat HCV^c^
Sample type	Plasma	Capillary blood	Plasma	Plasma, capillary blood
Sensitivity, %	100	98	99	95
Specificity, %	97	99	100	99
Sample preparation	Centrifugation required, all subsequent steps are integrated	Fully integrated	Centrifugation required; additional off-board sample preparation steps (several pipetting steps)	Centrifugation required for testing from plasma; a separate device required for sample preparation (both plasma and whole blood); pipetting steps
Time to result, min	110	60	Approximately 120	60
Regulatory status	CE-IVD, WHO PQ	CE-IVD	CE-IVD	India
Power supply	Needs an electricity supply	Needs an electricity supply	Needs an electricity supply	Batteries
Data analysis	PC	PC	Integrated	Integrated

Abbreviations: WHO PQ, World Health Organization prequalification, CE-IVD, CE-marked *in vitro* diagnostics.

^a^ https://www.cepheid.com/en/tests/Virology/Xpert-HCV-Viral-Load; https://www.cepheid.com/en/tests/Virology/Xpert-HCV-VL-Fingerstick.

^b^ https://supply.unicef.org/s0003968.html.

^c^ https://www.molbiodiagnostics.com/product_details.php?id=22.

The UK-based GeneDrive has also developed a qualitative HCV assay that can detect HCV RNA in 30 μL of plasma [23]. The GeneDrive platform has a smaller footprint than the GeneXpert system, weighs only 500 g, and does not require an external computer for data analysis and readout. However, just like the GeneXpert, the platform requires an electricity supply to run the test. Importantly, the HCV ID assay cartridge is not fully integrated into the system and requires several manual steps and precise pipetting. The assay performance has been evaluated in several studies, including studies conducted in resource-limited settings and primary healthcare facilities [24], which demonstrated that the assay has a sensitivity of 99% (95% CI, 98%–100%) and a specificity of 100% (95% CI, 99%–100%) [20]. Unfortunately, despite its good performance, the product was recently discontinued.

India-based Molbio diagnostics has developed a chip-based rapid PCR assay for qualitative detection of HCV RNA (Truenat HCV). The assay can be performed using the Molbio cartridge-based automated universal extraction system, the Truepep AUTO. Trueprep AUTO extraction can be done from 250 μL of fingerstick whole blood (approximately 7 drops) or 500 μL of plasma or serum in less than 20 minutes. Six microliters of purified RNA is transferred to the Truenat HCV chip using an automatic pipette provided with the system; the test results are available in 35 minutes [20, 25]. Both the Truepep AUTO and Truenat devices are portable and have an integrated rechargeable battery. The assay performance has been evaluated in a multicenter clinical study conducted in Spain, Ukraine, Georgia, and Thailand, which reported an overall sensitivity of 95% (95% CI, 93%–96%) and a specificity of 99% (95% CI, 99%–100%) [20].

It is essential to highlight that the clinical performance assessment of the near-POC HCV assays mentioned above was conducted in tightly controlled environments, primarily within well-equipped laboratories, and executed by extensively trained research personnel. In real-world scenarios, particularly in high-throughput and resource-limited settings, the assay's performance could be influenced by elevated cross-contamination risks, inadequate operator training, and variable environmental conditions. Consequently, it is imperative to take into account not only the reported test performance from clinical evaluations but also the context in which these assays are being employed.

## TRUE POINT-OF-CARE TESTING PLATFORMS THAT COULD POTENTIALLY INTEGRATE HCV ASSAYS

The coronavirus disease 2019 (COVID-19) pandemic underscored the role of testing in primary care, community, and home settings, as well as the importance of having appropriate tools to conduct this testing. The market size for POC diagnostics grew exponentially in both size and value during this period [26], with significant investments towards the development of new platforms. These investments spurred innovation at a pace never seen before and revolutionized the true-POC technology landscape [27, 28]. Molecular detection methods that can now be integrated into miniaturized battery-powered platforms have expanded the use of nucleic acid amplification testing to nonclinical settings, and even in the home. Many of the new platforms entered the market with a single assay for detection of severe acute respiratory syndrome coronavirus 2 (SARS-CoV-2) RNA from nasal swabs. As the overall demand for COVID-19 testing has been declining, some companies have struggled to sustain their operations [29], while others are working to expand their assay menu to include testing for other diseases. In the majority of cases, detection of SARS-CoV-2 RNA from nasal swabs does not require complex sample preparation, and usually a simple chemical lysis method is performed. However, it is yet to be established whether the new true-POC technologies are compatible with more complex sample types such as whole blood; in some cases, an additional sample preparation unit may be needed [30]. Some examples of technologies that employ a simple, handheld, battery-operated instrument with heating and/or detection functions, together with assay-specific consumables, are listed in Table 2. These technologies illustrate different architectures of true-POC systems that could be potentially used for HCV testing and several manufacturers have an HCV assay in their development pipeline. Examples of technologies that are sometimes described as “instrument-free,” meaning that the entire platform is disposable and built to be compatible with home use and self-testing, are presented in Table 3. Currently, none of the existing instrument-free systems have blood detection capabilities, but they may have the potential to be used for capillary blood, and hence HCV testing, through addition of an external module.

**Table 2.** **True Point-of-Care Testing Platforms That Could Potentially Integrate HCV Assays** ^a^

	Nuclein, DASH^b^	Mirai Genomics, GenPad^c^	PlusLife, Mini Dock^d^	ThermoFisher Scientific, Accula^e^
Sample preparation	Chemical lysis, RNA filtering^f^	Chemical lysis, RNA filtering^g^	Thermal and chemical lysis	Thermal and chemical lysis
Amplification method	RT-qPCR	Smart Amp, proprietary isothermal technology	RHAM, proprietary isothermal technology	RT-PCR
Turnaround time, min	15	40	15–35	30
Tests menu, commercially available	SARS-CoV-2	SARS-CoV-2, SARS-CoV-2/Flu A/B	SARS-CoV-2, SARS CoV-2/Flu A/B, mpox (RUO)	SARS-CoV-2, Flu A/B
Tests in development	HCV, HIV, STDs, Flu	Strep A, STDs	HPV, HCV, *M. tuberculosis*, Strep A, STDs	NA

Abbreviations: Flu, influenza; HCV, hepatitis C virus; HIV, human immunodeficiency virus; HPV, human papillomavirus; *M. tuberculosis, Mycobacterium tuberculosis;* RHAM, RNAse H-dependent amplification; RT-PCR, reverse transcription polymerase chain reaction; RT-qPCR, reverse transcription quantitative polymerase chain reaction; RNA, ribonucleic acid; RUO, research use only; SARS-CoV-2, severe acute respiratory syndrome coronavirus 2; STDs, sexually transmitted diseases; Strep A, *Streptococcus* A; NA, information not available.

^a^The technologies selected are illustrative examples to demonstrate different types of testing systems. The information about test pipeline is taken from company websites.

^b^ https://www.nuclein.com/technology/.

^c^ https://miraigenomics.com/.

^d^ https://www.pluslife.com/.

^e^ https://www.thermofisher.com/order/catalog/product/D2000.

^f^Via paramagnetic particles.

^g^Via columns on the cartridge.

**Table 3.** **Instrument-Free Technologies With the Potential to be Used as HCV Assays** ^a^

	Visby Medical^b^	Sherlock Biosciences^c^	Ustar PortNAT^d^	Midge Medical Minoo^e^
Sample preparation	Thermal and chemical lysis	Thermal and chemical lysis	Thermal and chemical lysis	Thermal and chemical lysis
Amplification method	RT-PCR	Isothermal/CRISPR	Proprietary isothermal amplification technology	RPA
Turnaround time, min	30	15	25	NA
Tests menu, commercially available	SARS-CoV-2, Flu A/B; STDs	NA	SARS-CoV-2	NA
Power supply	Needs electricity	Battery powered	Battery powered	Battery powered
Tests in development	NA	STDs, respiratory diseases	HIV, CT/NG, Flu	SARS-CoV-2, HIV, *M. tuberculosis*, Flu, herpes virus, Ebola virus

Abbreviations: CRISPR, clustered, regularly interspaced, short palindromic repeat; CT/NG, *Chlamydia trachomatis*, *Neisseria gonorrhea, Trichomonas vaginalis;* Flu, influenza; HCV, hepatitis C virus; HIV, human immunodeficiency virus; *M. tuberculosis, Mycobacterium tuberculosis;* RPA, recombinant polymerase amplification; RT-PCR, reverse transcription polymerase chain reaction; SARS-CoV-2, severe acute respiratory syndrome coronavirus 2; STDs, sexually transmitted diseases.

^a^The technologies selected are illustrative examples to demonstrate different types of testing systems. The information about test pipeline is taken from company websites.

^b^ https://www.visbymedical.com/sexual-health-test/.

^c^ https://sherlock.bio/platforms/crispr/.

^d^ https://en.bioustar.com/product/155.html.

^e^ https://www.midgemedical.com/.

It is important to note that most true-POC platforms rely on isothermal amplification. A number of research articles have reported the feasibility of different isothermal methods for HCV RNA detection. Wang et al showed that reverse transcription recombinase-aided amplification can detect HCV RNA in 30 minutes [31], and Chia et al demonstrated the applicability of RPA for HCV RNA detection [32]. Although isothermal methods were tested in laboratory settings and not on an integrated POC device, available data can be considered as a proof-of-concept, indicating that development of HCV assays on a true-POC platform using isothermal methods may be possible.

## PROMISING FUTURE TECHNOLOGIES

The challenge of conducting affordable and accurate molecular diagnosis from blood can potentially be tackled by new upcoming technologies. The requirement for sample preparation and genetic material extraction from blood may limit the performances of existing isothermal-based approaches, as they may lead to low specificity. In this context, isothermal amplification methods combined with clustered, regularly interspaced, short palindromic repeat (CRISPR)-based detection holds promises for improving clinical sensitivity and specificity. CRISPR-based readouts can be combined with LAMP or other isothermal amplification method to increase assay specificity, mostly by reducing the signal of the negative samples. High accuracy of HCV detection using a CRISPR-based readout combined with LAMP amplification has been recently demonstrated [33].

As shown in Table 3, Sherlock Biosciences is an example of a company working toward implementation of this approach on a true-POC format. Several studies have demonstrated an effective implementation of a CRISPR-based assay on a lateral flow strip, and this could lead to a significant cost reduction in disposable tests. Furthermore, it has recently been demonstrated that HCV RNA extracted from clinical samples can be successfully detected using reverse transcriptase (RT)-LAMP amplification combined with CRISPR [33].

Biosensor-based approaches based on semiconductor technology or micro-electro-mechanical system (MEMS) sensors represent an alternative approach to reducing turnaround time and the overall device cost [34, 35]. Several research groups and companies are also exploring the potential of graphene-based sensors for POC diagnostics [36]. Cardea Bio and Identify Sensors are developing a graphene-based biosensing solution that may not require target sequence amplification thanks to the intrinsically high platform sensitivity. However, the development of robust assays over this type of platform has still to be fully demonstrated and will need to overcome various challenges, particularly related to controlling the interaction of charged molecules and buffer composition to ensure compatibility with the sensors.

## THE ROLE OF EXISTING AND NEW HCV DIAGNOSTICS IN BEST PRACTICE

It is clear that emerging HCV true-POC technologies may help further HCV elimination efforts, particularly among marginalized populations and hard-to-reach communities with limited access to centralized healthcare and high loss to follow-up [4]. A systematic review of 45 studies has shown that using near-POC HCV RNA assays instead of a centralized laboratory-based approach improved the efficiency of HCV programs, resulting in a quicker turnaround time between testing and treatment (19 days vs 64–66 days) and a 32% increase in treatment uptake [37].

Employing a completely decentralized one-stop-shop model (where the patient only attends one low-level health facility for all diagnosis and treatment needs) could further increase linkage to care and treatment, particularly in high-risk groups such as PWID and incarcerated persons. A systematic review of 142 studies, including nearly half a million patients from LMICs, showed that this approach led to successful linkage to care in 72% of PWID and 94% of incarcerated persons, compared with 53% and 50%, respectively, for approaches that required the patient to move from one health facility to another [38]. Similarly, treatment uptake was higher with full decentralization compared with partial decentralization (73% vs 66% and 72% vs 39% for PWID and prisoners, respectively) [38]. This study supports the findings of studies in India and Malaysia, which showed that significantly fewer patients were lost to follow-up during the diagnostic and treatment cascade if all services were provided at a single site (vs referral to a different site/hospital), resulting in reduced loss to follow-up between screening and confirmatory testing, higher rates of treatment, and shorter turnaround times [39, 40]. Similarly, a prospective study in the United States revealed that a significantly greater proportion of HCV-seropositive PWID received viremic testing in a POC setting or at a harm reduction site (where venous blood samples were collected for off-site testing) compared with the referral of patients between sites for blood collection and/or testing [41]. These studies, combined with the high retention rates also observed across the care cascade [41], highlight the importance of conducting as much testing as possible at a single site [42]. True-POC technologies for HCV could enable expansion of one-stop-shop facilities and decentralization of HCV care to primary healthcare clinics, pharmacies, and harm reduction sites, by enabling an expansion of on-site HCV diagnosis to complement existing on-site treatment.

Regarding the diagnostic landscape, liver testing also needs to be considered alongside HCV testing. This is essential in hepatitis C as it helps determine the appropriate treatment and posttreatment follow-up. For example, the WHO recommendation on treatment duration varies from 8 to 24 weeks, dependent on the presence of compensated cirrhosis, the HCV genotype, and the type of treatment (sofosbuvir/daclatasvir or glecaprevir/pibrentasvir) [4]. There is currently no POC liver staging, therefore, even if true-POC for HCV RNA was developed, same-day treatment initiation may not be possible. Coupling true-POC HCV testing with POC liver staging could be considered best practice—and would be in line with current WHO guidelines, which recommend liver staging prior to treatment initiation [4]. However, if a diagnostic and treatment algorithm involves ruling out decompensated cirrhosis using clinical signs, then starting treatment solely based on detection of HCV RNA while awaiting confirmation of liver staging results, may also be an appropriate alternative.

Given that there is no international funding body for hepatitis C as there is for human immunodeficiency virus (HIV), tuberculosis, or malaria, budgets for HCV programs are often limited. As such, governments will need to make informed choices about optimal diagnostic algorithms. Cost-effectiveness research has shown that skipping HCV antibody testing and going straight to HCV viremia testing may be cost-effective, but only in populations with a very high viremia prevalence rate (46.9% and above) and only if the cost of HCV RNA testing is less than €7.32 per test [43]. Currently available near-POC are not meeting this price target and hence are not suitable for a 1-step approach. It is possible that with the new larger pipeline of near-POC and true-POC technologies, cheaper and faster tests will become available and eventually enable a 1-step approach in high-risk populations.

Overall, depending on the target population and current facilities available, both near-POC and true-POC platforms could be effectively deployed to complement centralized laboratory testing. Implementing these platforms in the healthcare settings where they are needed and can be used would allow provision of timely linkage to care and expand access to HCV testing to currently underserved populations. In addition, it is important to note that care provision should be holistic and include other health service provision such as testing and linkage to care for hepatitis B surface antigen (HBsAg) and HIV.

## Notes

***Acknowledgments.*** The authors acknowledge Laura Mazzola (FIND) for her assistance in the development of Figure 1 and preparation of the manuscript. Editorial assistance in the preparation of the manuscript was also provided by Stuart Wakelin.

***Author contributions.*** All authors were involved in the drafting and revising of the manuscript and approved the final version for submission.

***Supplement sponsorship.*** This article appears as part of the supplement “Towards a Single-Step Diagnosis of Hepatitis C Virus Infection,” sponsored by the Centers for Disease Control and Prevention.

***Financial support*.** None.
